# Complications associated with endobronchial ultrasound-guided transbronchial needle aspiration: a nationwide survey by the Japan Society for Respiratory Endoscopy

**DOI:** 10.1186/1465-9921-14-50

**Published:** 2013-05-10

**Authors:** Fumihiro Asano, Motoi Aoe, Yoshinobu Ohsaki, Yoshinori Okada, Shinji Sasada, Shigeki Sato, Eiichi Suzuki, Hiroshi Semba, Kazuya Fukuoka, Shozo Fujino, Kazumitsu Ohmori

**Affiliations:** 1 Safety Management Committee, Japan Society for Respiratory Endoscopy, Tokyo, Japan; 2 Department of Pulmonary Medicine, Gifu Prefectural General Medical Center, 4-6-1 Noishiki, Gifu 500-8717, Japan

**Keywords:** Breakage, Bronchoscopic ultrasound, Bronchoscopy, Infection, Lung cancer, Mediastinitis, Mortality, Pulmonary intervention, Safety, Survey

## Abstract

**Background:**

With the recent widespread use of endobronchial ultrasound-guided transbronchial needle aspiration (EBUS-TBNA), there have been occasional reports on complications associated with its use. Previous reviews on EBUS-TBNA have been limited to studies by skilled operators, thus the results may not always be applicable to recent clinical practice. To assess the safety of EBUS-TBNA for the staging and diagnosis of lung cancer in Japan, a nationwide survey on its current usage status and complications associated with its use was conducted by the Japan Society for Respiratory Endoscopy (JSRE).

**Methods:**

A questionnaire about EBUS-TBNA performed between January 2011 and June 2012 was mailed to 520 JSRE-accredited facilities.

**Results:**

Responses were obtained from 455 facilities (87.5%). During the study period, EBUS-TBNA was performed in 7,345 cases in 210 facilities (46.2%) using a convex probe ultrasound bronchoscope, for 6,836 mediastinal and hilar lesions and 275 lung parenchymal lesions. Ninety complications occurred in 32 facilities. The complication rate was 1.23% (95% confidence interval, 0.97%-1.48%), with hemorrhage being the most frequent complication (50 cases, 0.68%). Infectious complications developed in 14 cases (0.19%) (Mediastinitis, 7; pneumonia, 4; pericarditis, 1; cyst infection, 1; and sepsis, 1). Pneumothorax developed in 2 cases (0.03%), one of which required tube drainage. Regarding the outcome of the cases with complications, prolonged hospitalization was observed in 14 cases, life-threatening conditions in 4, and death in 1 (severe cerebral infarction) (mortality rate, 0.01%). Breakage of the ultrasound bronchoscope occurred in 98 cases (1.33%) in 67 facilities (31.9%), and that of the puncture needle in 15 cases (0.20%) in 8 facilities (3.8%).

**Conclusions:**

Although the complication rate associated with EBUS-TBNA was found to be low, severe complications, including infectious complications, were observed, and the incidence of device breakage was high. Since the use of EBUS-TBNA is rapidly expanding in Japan, an educational program for its safe performance should be immediately established.

## Background

Endobronchial ultrasound-guided transbronchial needle aspiration (EBUS-TBNA) performed using a convex probe ultrasound bronchoscope is a minimally invasive diagnostic method for peritracheal and peribronchial lesions [1]. EBUS-TBNA enables the accurate collection of samples from the lesion since the puncture site can be confirmed under real-time ultrasound guidance, allowing cytological and histological diagnosis. In the lymph node staging of lung cancer, EBUS-TBNA has a cumulative sensitivity of 88%-93% and a cumulative specificity of 100% [2,3]. For the diagnosis of mediastinal lymph node metastasis of lung cancer, mediastinoscopy is conventionally considered as the gold standard [4]. However, compared with surgical diagnosis using a mediastinoscope or thoracoscope, EBUS-TBNA and transesophageal endoscopic ultrasound with fine needle aspiration (EUS-FNA) followed by surgical diagnosis have been reported to increase the diagnostic rate [5]. EBUS-TBNA alone has a diagnostic rate similar to that of mediastinoscopy [6,7]. In addition, the introduction of EBUS-TBNA has resulted in cost benefit in terms of minimally invasive lung cancer staging [8,9]. Hence, EBUS-TBNA has effectively been replacing mediastinoscopy for the staging of lung cancer and in establishing a definitive diagnosis of mediastinal and hilar lymphadenopathy owing to its high diagnostic rate, minimal invasiveness, and cost benefit. EBUS-TBNA has also an extremely low complication rate and is therefore considered to be safe and minimally invasive [2,10].

However, there have been occasional reports on complications with the recent widespread use of EBUS-TBNA [11-14]. Moreover, previous reviews on EBUS-TBNA have been limited to studies by skilled operators, and thus the results may not always be applicable to recent clinical practice. Previous reviews particularly on the cost benefit of EBUS-TBNA have not included the repair cost for damaged EBUS-TBNA instruments. As EBUS-TBNA will increasingly be used for the staging and diagnosis of lung cancer, accompanying severe complications may also increase. Damage to EBUS-TBNA instruments not only poses a problem with regard to the cost benefit, but also threatens the safety and effectiveness of the procedure. To assess the safety of EBUS-TBNA for the staging and diagnosis of lung cancer in Japan, a nationwide survey on its current usage status and complications associated with its use was conducted by JSRE.

## Methods

This study was a nationwide, retrospective questionnaire survey approved by JSRE. In July 2012, a JSRE-approved questionnaire was mailed to 520 JSRE-accredited facilities and affiliated facilities throughout Japan. The questionnaire (an additional data file shows this in more detail [see Additional file 1]) included specific questions regarding the number of cases examined by EBUS-TBNA, the characteristics of the cases, and the complications of EBUS-TBNA performed using a convex probe ultrasound bronchoscope during the preceding 18 months (January 2011 through June 2012). Details regarding the complications were obtained by asking questions about patient background, procedural factors, and outcomes using an exclusive case report form. A mark sheet selection system was used, except for some items that were to be described using free text. As a rule, a JSRE-accredited senior fellow representing each institution provided the responses to the questionnaire. The number of cases, number of cases with complications, and details of cases showing complications were reported by the same senior fellow, based on the medical records in each institution. The investigated complications included hemorrhage that required treatments other than aspiration, compression and cold physiological saline injection (massive hemorrhage was defined as a blood loss of ≥ 300 mL or that requiring blood transfusion), pneumonia, mediastinitis, pericarditis, other infectious complications, pneumothorax requiring bed rest or thoracic drainage, lidocaine intoxication requiring special intervention because of convulsions/loss of consciousness, bronchial asthma, respiratory failure (excluding patients treated by oxygen administration alone), and complications related to the circulatory system requiring special treatment, among others. Complications due to procedures performed simultaneously with EBUS-TBNA, such as forceps biopsy or bronchoalveolar lavage, were excluded.

Results were automatically collected using a computer. For multiple-choice questions permitting only 1 response, no response to the question was regarded as an invalid response. All proportions were expressed as a percentage of the number of valid responses. Results were analyzed using a standard statistical method with the SAS system for Windows release 9.3 (SAS Institute Inc., Cary, NC, USA).

## Results

Responses were obtained from 455 facilities (response rate, 87.5%).

### Number of cases

EBUS-TBNA performed using a convex probe was carried out in 7,345 cases in 210 facilities (46.2%) out of the 455 facilities that responded. The indications of the procedure (multiple selections allowed) were for the establishment of a diagnosis in 5,855 cases (suspected lung cancer in 4,304 cases, suspected malignant lymphoma in 321 cases, suspected sarcoidosis in 988 cases, and others in 527 cases; multiple selections allowed) and the staging of lung cancer in 1,850 cases. The lesion was located in the hilar and mediastinal areas in 6,836 cases and in the lung parenchyma in 275 cases (Table 1). EBUS-TBNA was performed by JSRE-accredited non-fellows in 3,442 cases (46.9%) and JSRE-accredited fellows in 3,896 cases (53.1%); no information regarding the operators was given in 7 cases.

**Table 1:** Total number of EBUS-TBNA procedures performed according to indication and target lesion

	**Total number**	**Respondents**	**Mean**	**Median**	**Range**
Total EBUS-TBNA	7,345	210	35.0	18.5	1-424
*Indication:*					
Establishing diagnosis	5,855	207	28.3	16	1-223
Lung cancer staging	1,850	111	16.7	5	1-245
*Lesions examined:*					
Hilar and mediastinal lesions	6,836	209	32.7	17	1-235
Lesions in lung parenchyma	275	68	4.0	2	1-22

EBUS-TBNA, endobronchial ultrasound-guided transbronchial needle aspiration.

### Complications

Complications developed in 90 cases (1.23%; 95% confidence interval (CI), 0.97%-1.48%) in 32 facilities (15.2%). For complications, hemorrhage was observed in 50 cases (0.68%; 95% CI, 0.49%-0.87%) including 1 case of massive hemorrhage. Mediastinitis developed in 7 cases (0.10%; 95% CI, 0.02%-0.17%), pneumonia in 4 cases (0.05%), pericarditis in 1 case (0.01%), cyst infection in 1 case (0.01%), and sepsis in 1 case (0.01%). Infectious complications (mediastinitis, pneumonia, pericarditis, cyst infection and sepsis) occurred in 14 cases (0.19%; 95% CI, 0.09%-0.29%). Respiratory failure developed in 5 cases (0.07%). Lidocaine toxicity was observed in 4 cases (0.05%). Pneumothorax was observed in 2 cases (0.03%), 1 of which required tube drainage. Bronchial asthmatic attack was observed in 1 case (0.01%). As for cardiovascular events, arrhythmia developed in 3 cases (0.04%) and hypotension in 1 case (0.01%). Regarding other complications, fever was observed in 4 cases (0.05%), cerebral infarction in 2 cases (0.03%), aggravation of airway obstruction in 2 cases (0.03%), tumor rupture in 1 case (0.01%), and hyperventilation syndrome in 1 case (0.01%).

### Reports of cases with complications

There were reports of 79 cases with complications. The background, procedural factors, and outcomes of these cases are described below.

### Patient background

The median age of the patients was 69 years (25–87 years). There were 59 men (75.6%) and 19 women (24.4%), with 1 patient whose sex was undisclosed. The targets for puncture were the hilar and mediastinal lymph nodes in 76 cases (96.2%) and lesions in the lung parenchyma in 3 cases (3.8%). The complication rates were 1.11% and 1.09%, respectively, with no statistically significant difference in the complication rates between the target lesions (p > 0.999; Fisher exact test).

### Background of operators

The operators were JSRE-accredited non-fellows in 46 cases (58.2%) and JSRE-accredited fellows in 33 cases (41.8%). The complication rate was 1.34% for JSRE-accredited non-fellows and 0.85% for JSRE-accredited fellows (p = 0.0535; Fisher exact test), being slightly higher in the former. In the patients who developed EBUS-TBNA-related complications, the operators had the following EBUS-TBNA experience before the occurrence of complications: fewer than 5 cases in 8 patients (10.3%), 5–19 cases in 17 patients (21.8%), 20–49 cases in 43 patients (55.1%), and more than or equal to 50 cases in 10 patients (12.8%); there was no description regarding the background of the operator in 1 case. The operators had attended a hands-on training course in 41 cases (52.6%) and had not attended a hands-on training course in 37 (47.4%) cases; there was no information whether the operator attended a hands-on training course in 1 case.

### Procedural factors

Sixty-once cases received intravenous sedation (79.2%) whereas 16 cases (20.8%) did not receive intravenous sedation during the procedure. There was no information regarding intravenous sedation during the procedure for 2 cases. A 22-gauge needle was used in 53 cases (76.8%) and a 21-gauge needle was used in 16 cases (23.2%); there was no description about the needle gauge used in 10 cases. The number of lymph nodes punctured was 1 in 59 cases (76.6%) and 2 in 18 cases (23.4%); there was no information about puncture number in 2 cases. The maximum short-axis diameter of the punctured lymph node was from 5 to < 10 mm in 4 cases (5.8%) and ≥ 10 mm in 65 cases (94.2%); there was no description given in 10 cases. The most frequently punctured lymph node was #7 in 34 patients (43.0%), followed by #4R in 24 patients (30.4%). The total number of punctures was 1 in 32 cases (41.6%), 2 in 26 cases (33.8%), 3 in 9 cases (11.7%), 4 in 9 cases (11.7%), and ≥ 5 in 1 case (1.3%); there was no description available in 2 cases. On-site cytodiagnosis was performed in 27 cases (35.1%) and not performed in 50 cases (64.9%); there was no description given in 2 cases.

On the basis of the EBUS-TBNA findings, a specific diagnosis could be made in 58 cases (76.3%) but not in 18 cases (23.7%; non-diagnostic cases: diagnosis could not be made based on the EBUS-TBNA findings); there was no description provided in 3 cases. The final diagnosis based on the surgical findings and clinical course, in addition to the EBUS-TBNA findings, was lung cancer in 49 cases (66.2%), metastatic lung tumor in 3 cases (4.1%), malignant lymphoma in 2 cases (2.7%), other malignant tumors in 3 cases (4.1%), sarcoidosis in 3 cases (4.1%), other benign diseases in 8 cases (10.8%), and indefinite (the final diagnosis could not be made using any methods including EBUS-TBNA or observation of the clinical course) in 6 cases (8.1%); there was no description given in 5 cases.

### Outcomes

Complication-induced adverse events were absent in 57 cases that developed complications (75.0%). Hospitalization was prolonged in 14 cases (18.4%) because of pneumonia in 4 cases, respiratory failure in 3 cases, hemorrhage in 2 cases, mediastinitis in 1 case, pneumothorax in 1 case, bronchial cyst infection in 1 case, fever in 1 case, and cerebral infarction in 1 case. Life-threatening consequences of the following complications were observed in 4 cases (5.3%): mediastinitis, 2 cases; tumor rupture, 1 case; and aggravation of airway stenosis, 1 case. Death due to cerebral infarction was observed in 1 case (1.3%). The mortality rate was 0.01%. There was no description of adverse events in 3 cases.

### Characteristics of each complication

Hemorrhage was observed in 42 cases, of which 1 patient showed massive hemorrhage. In this case, hemorrhage after the puncture of lymph node #11L, possibly due to damage of the blood vessels, required prolongation of hospitalization. None of the 42 cases were being treated with antiplatelet drugs; 4 cases (22.2%) were in a drug withdrawal period, and no antiplatelet drug had been used in 14 cases (77.8%). There was no description available in 24 cases.

The maximum short-axis diameter of the punctured lymph node was from 5 to <10 mm in 2 cases (5.9%) and ≥10 mm in 32 cases (94.1%); there was no description regarding this in 8 cases. The most frequently punctured lymph node was #7 in 34 patients (43.0%), followed by #4R in 24 patients (30.4%). The total number of punctures was 1 in 24 cases (57.1%), 2 in 14 cases (33.3%), 3 in 1 case (2.4%), and 4 in 3 cases (7.1%). A 22-gauge needle was used in 30 cases (85.7%) and a 21-gauge needle was used in 5 cases (14.3%) with hemorrhage; there was no description given in 7 cases.

The operator was a JSRE-accredited non-fellow in 26 cases (61.9%) and a JSRE-accredited fellow in 16 cases (38.1%). In patients who developed EBUS-TBNA-related complications, the operators had the following EBUS-TBNA experience before the occurrence of complications: 5–19 cases in 5 patients (11.9%), 20–49 cases in 32 patients (76.2%), and ≥ 50 cases in 5 patients (11.9%).

Mediastinitis was observed in 7 cases, of which only 1 had diabetes mellitus as an underlying disease. The punctured lymph node was #4R in 4 and #7 in 3 of the mediastinitis cases. Ultrasound findings at the puncture site of the 7 cases suggested necrotic areas in 2 cases (33.3%), other than necrosis or cysts in 2 cases (33.3%), and unclear in 2 cases (33.3%); there was no description in 1 case. In 11 (84.6%) of 14 cases showing infectious complications, no prophylactic antibiotic had been administered.

In the 2 cases developing pneumothorax, 5 punctures of lymph nodes #4R and #7 and 4 punctures of lymph node #4L had been performed. In a case showing asthmatic attacks, although there was a history of bronchial asthma, no prophylactic bronchodilator had been administered before examination. Two cases showed cerebral infarction during the antiplatelet drug withdrawal period. One of these cases showed bilateral stenosis of the internal carotid arteries and replacement of the anticoagulant with heparin. This patient died of thrombotic occlusion of these arteries after completion of the examination. The case of tumor rupture, which was confirmed by surgery, involved a mediastinal tumor in which pleural hemorrhage occurred after EBUS-TBNA.

### Breakage of EBUS-TBNA instruments

During the investigation period, the ultrasound bronchoscope used was damaged in 98 cases (1.33%; 95% CI, 1.07%-1.60%) in 67 facilities (31.9%) (Table 2). Regarding the damaged part, breakage of the working channel for needle insertion was found in 71 cases (74.0%), breakage of the fiber owing to external compression, such as biting by the patient, occurred in 15 cases (15.6%), breakage of the ultrasound probe in 7 cases (7.3%), and breakage of other portions in 3 cases (3.1%); there was no description available in 2 cases. Breakage of the puncture needle occurred in 15 cases (0.20%; 95% CI, 0.10%-0.31%) in 8 facilities (3.8%).

**Table 2:** Breakage of EBUS-TBNA instruments and reasons for these breakages

	**Total number**	**Respondents**	**Mean**	**Median**	**Range**
*Site of damage:*					
Bronchoscopes	98	67	1.46	1	1-5
Working channel	71	50	1.42	1	1-4
Fiber portion	15	15	1	1	1
US probe	7	7	1	1	1
Other portions	3	2	1.33	1	1-2
Puncture needle	15	8	1.63	1	1-4

EBUS-TBNA, endobronchial ultrasound-guided transbronchial needle aspiration; US, ultrasound.

## Discussion

To the best of our knowledge, this is the first nationwide survey describing in detail the complications associated with the use of EBUS-TBNA. As this survey was administered by a scientific society, and the obtained response rate to the questionnaire was considerably high (87.5%), the results may effectively reflect the present status of the use of and complications associated with EBUS-TBNA in Japan. A previous questionnaire survey performed in Japan in 2010 showed that 143 facilities nationwide owned and used EBUS-TBNA devices [15]. This number increased by 67 during the subsequent 1.5-year period. The number of cases examined using EBUS-TBNA per year, as calculated from the number of questionnaires recovered, was 4,108 cases in a previous survey and 5,596 in the present survey, indicating an approximately 1.4-fold increase in the number of EBUS-TBNA performed per year.

Previous meta-analyses of conventional TBNA showed a major complication rate of 0.3% [16]. Massive hemorrhage, pneumomediastinum, pneumothorax requiring drainage, cardiac tamponade, and hemomediastinum have all been reported as TBNA complications [16,17]. A meta-analysis of EBUS-TBNA showed a complication rate of 2 in 1,299 cases (0.15%), with only 1 case showing pneumothorax requiring drainage as a major complication [2]. However, the studies evaluated by this meta-analysis had been performed in facilities with skilled operators. With the recent widespread use of EBUS-TBNA, complications have occasionally been reported [11-14]. A survey in 2010 showed a complication rate of 0.46% for EBUS-TBNA [18]. On the other hand, a prospective study by Eapen et al. revealed a complication rate of 1.44% [19]. The incidence of complications differs and depends not only among medical examination/treatment environments, but also on the definitions of the complications. The definitions of the different complications assessed in this study were the same as those in the 2010 survey, except for hemorrhage. In the present survey, a broader definition of hemorrhage was used compared with the previous survey, and hemorrhage that required treatment was also included in the list of complications. This possibly accounted for the higher complication rate in the present study. Furthermore, the incidence of the different types of complications in the present survey differed from that reported by Eapen et al. [19]. In the present survey, the incidence of pneumothorax was low but that of infectious complications was high. This may have been due to not only differences in the medical examination/treatment environments between Japan and the U.S. as well as differences in the survey methods, but also the evaluation of only complications developing within 24 hours and the inclusion of complications due to other procedures such as transbronchial biopsy that were simultaneously performed in the survey by Eapen et al. The complication rate in this survey was lower than that using mediastinoscopy (2.5%) [20] or other bronchoscopic diagnostic methods [18], demonstrating the safety of EBUS-TBNA.

However, in the present study, death or life-threatening consequences were observed in 5 cases. In addition, the incidence of infectious complications was high. Regarding infectious complications, mediastinitis [12,13], pericarditis [11], and tumor infection [11] have been reported, each of which is a severe condition requiring broad-spectrum antibiotics and surgical procedures such as drainage. Two cases in the present study were considered to have life-threatening conditions. As for the possible causes of infectious complications, cross-contamination, leading to mediastinal inoculation by oral and nasopharyngeal commensals [11,13], and nosocomial infection due to inappropriate bronchoscope disinfection or mishandling of devices [12] have been reported. Previous studies have suggested that these infectious complications are not caused by hematogenous seeding due to bacteremia, but by spread from the puncture site [13]. When the puncture site is necrotic or cystic, blood flow is slight, resistance is decreased, and bacterial attachment clearance is decreased [11]. To prevent this condition, the location of the needle tip should be confirmed by ultrasonography at the time of puncture, and the lesion should be accurately punctured [11]. As in EUS-FNA, the puncture of necrotic [21] or cystic areas [22] should be avoided. Unlike conventional TBNA, lesions are punctured with a puncture needle containing a stylet in EBUS-TBNA. Since infection may be caused by operating the stylet, it should be handled aseptically when multiple punctures are applied. Disinfection of devices according to the guidelines [23] and careful observation of the postprocedure course for signs of infection are important. However, prophylactic antibiotic administration is not recommended in routine diagnostic bronchoscopy, except for certain diseases [24]. In the present study, there were no clearly immunocompromised patients with infectious complications, and prophylactic antibiotics were not administered in 11 of the 14 cases showing infectious complications. Further studies are necessary to evaluate the need for prophylactic antibiotic administration and to identify the cases requiring such prophylaxis. In particular, Eapen et al. reported patients who died of hemorrhage [19]. In the present study, only 1 case showed massive hemorrhage caused by perforation of a minor blood vessel in the lymph node. Botana-Rial et al. reported a case of erroneous puncture of the pulmonary artery [14] without symptoms and detected by echography or contrast CT. Thus, the incidence of erroneous puncture of a major vessel may be higher than that of massive hemorrhage as a complication. Hence, it is important to know the anatomy around the puncture area and to accurately puncture only the lesion. Bronchial asthma, cerebral infarction, and lidocaine intoxication were considered to be complications of the bronchoscopic procedure. In patients at risk, procedures following the guidelines are necessary, including prophylactic bronchodilator administration, continuation of antiplatelet drugs/anticoagulants or drug replacement depending on the underlying diseases and drug composition, adequate assessment of the indications for examination, and observance of the maximum lidocaine dose that can be safely administered [24].

In the present study, the complication rate was slightly higher in the procedures performed by JSRE-accredited non-fellows than in those performed by JSRE-accredited fellows. Regarding the diagnostic rate, a prospective study previously showed that high-volume hospitals had high diagnostic yields, and that biopsy was performed on more nodes including smaller nodes [25]. Although Eapen et al. stated that safety is not associated with experience [19], their data were, however, obtained from tertiary care hospitals. Therefore, we cannot exclude the possibility that the complication and diagnosis rates differ among facilities and according to the operator’s experience. In EBUS-TBNA, the learning speed varies even among skilled bronchoscopists [26]. Moreover, the number of cases that must be performed to be able to carry out EBUS-TBNA safely and to achieve an adequate diagnostic rate is difficult to determine. The guidelines of the American College of Chest Physicians [27], European Respiratory Society/American Thoracic Society [28], and Thoracic Society of Australia and New Zealand [29] indicate that 50 (radial probe), 40 (convex and radial probe), and 20 cases, respectively, are necessary for this purpose, and that EBUS-TBNA must be performed in 25, 20, and 20 cases per year, respectively, to maintain EBUS-TBNA competency. The present survey did not include the number of patients undergoing EBUS-TBNA by operators with no experience of complications, and thus the complication rate according to the number of cases experienced by the operators could not be determined. Accordingly, the number of cases experienced which are necessary to acquire the skill to safely perform EBUS-TBNA is unclear. Most of the operators in the present study had experienced less than 50 EBUS-TBNA cases at the time of the occurrence of complications, and about 50% of the operators had never attended any hands-on training. At present, concrete instructions and educational methods are left to the judgment of each institution, and they vary among facilities. Experienced bronchoscopists, such as JSRE-accredited fellows in each institution, should receive hands-on training in the course authorized by JSRE in order to become supervisors of EBUS-TBNA. A common learning system [30] and an evaluation method across facilities using objective parameters of skills [31] should be immediately established and applied to enable EBUS-TBNA operators to receive optimal training under the instruction of qualified supervisors. In addition, useful simulation trainings [32] and their active incorporation into the learning system should also be considered.

With regard to device breakage, this has been infrequently reported [33]. In the present study, the breakage rate for EBUS-TBNA bronchoscopes and devices was 1.54%, which was significantly higher (p < 0.001, Fisher exact test) than that in all bronchoscopy procedures (0.77%) in the 2010 survey [18]. Breakage most commonly occurred in the working channel mostly due to the needle perforation of the working channel. Proper placement of the puncture needle in the sheath and its blockage should be confirmed before insertion and removal of the puncture needle. Tremblay et al. reported that the repair cost of EBUS-TBNA instruments for each patient is higher than that of the bronchoscope used for conventional flexible bronchoscopy. In the present survey, EBUS-TBNA was not performed in 245 facilities, possibly because of the lack of EBUS-TBNA instruments in most of these facilities [15]. Unlike conventional TBNA, EBUS-TBNA is complex and requires hands-on training. Moreover, EBUS-TBNA instruments are expensive, and their maintenance and repair costs are high because of their high damage rate, which may be obstacles to the introduction of EBUS-TBNA.

The present study has several limitations. Recall bias was highly likely because the study was a retrospective questionnaire survey. Some data may have been provided based on the memory of the persons who completed the questionnaire and thus some values may be inaccurate. Moreover, the surveyed subjects may have hesitated to report complications and deaths. Although the response rate to the questionnaire was high, the facilities with a high complication rate may not have participated. In Japan, chest radiography is usually performed following bronchoscopy to confirm the presence of pneumothorax [15]. However, the follow-up method to determine the occurrence of complications is entrusted to each facility and is not standardized. Accordingly, it is possible that asymptomatic complications and severe cases treated at other facilities were overlooked. Moreover, the present survey involved facilities certified by JSRE, and expert operators were included. Therefore, the complication rate may have been underestimated compared with the actual situation. Furthermore, when the complication and damage rates were combined, some problems occurred in more than 3% of the patients who received EBUS-TBNA.

## Conclusion

Although the complication rate for EBUS-TBNA was found to be low, severe complications and a high incidence of device breakage were observed during the study period. With the rapid and widespread use of EBUS-TBNA as a diagnostic method, an educational system providing clear instructions on how to properly perform the procedure safely needs to be immediately established.

## Abbreviations

EBUS-TBNA: Endobronchial ultrasound-guided transbronchial needle aspiration; EUS-FNA: Transesophageal endoscopic ultrasound with fine-needle aspiration; JSRE: The Japan Society for Respiratory Endoscopy; TBNA: Transbronchial needle aspiration.

## Competing interests

The authors declare that they have no competing interests associated with this study.

## Authors’ contributions

All authors contributed equally to the study design, data acquisition, and data analysis and interpretation. FA drafted the manuscript. All authors revised the manuscript critically for important intellectual content and gave their approval for it to be published.

## Supplementary Material

Additional file 1 **Questionnaire.** Nationwide Survey of EBUS-TBNA. Click here for file
