# Supplementary material for: Complications associated with endobronchial ultrasound-guided transbronchial needle aspiration: a nationwide survey by the Japan Society for Respiratory Endoscopy
Source: Respir Res. 2013 May 10;14(1):50. doi: 10.1186/1465-9921-14-50 (PMC3655828; doi:10.1186/1465-9921-14-50)
Supplement: Additional file 1 — Questionnaire. Nationwide Survey of EBUS-TBNA. [file 1465-9921-14-50-S1.doc]

**Questionnaire**

***Committee for the Nationwide Survey of EBUS-TBNA***

**Nationwide Survey of EBUS-TBNA**

Please provide your answers in this questionnaire regarding patients who underwent endobronchial ultrasound-guided transbronchial needle aspiration (EBUS-TBNA) (limited to the use of an ultrasonic bronchoscope) during the 1-year and 6-month period from January 1, 2011 to June 30, 2012 (survey period).

* When several departments perform bronchoscopy within your institution, please have a representative collectively fill in the form and send it back.

* Choose one answer to the questions unless multiple answers are allowed.

* Please enter ‘0’ when there is no corresponding case.

* **The submission deadline is August 31, 2012 (postmarked).**

Facility number

**State and experience of EBUS-TBNA of facility**

Was EBUS-TBNA performed at your facility during the survey period: Yes/No

If your answer is ‘No’, is there is a plan to introduce EBUS-TBNA in your institution: Yes/No

Name of person who filled in this questionnaire ( )

Affiliated department ( )

Institution ( )

Contact e-mail address ( )

**Survey of the current state of EBUS-TBNA use**

*Important notes on answering the questions*

***Operator*** is defined as the physician who performed the puncture.

Exclude complications assumed to be caused by procedures other than EBUS-TBNA, such as forceps biopsy and alveolar lavage.

When 2 or more complications were caused by a single examination, enter those in individual columns of complications.

When there is no corresponding case, enter ‘0’.

Complications are defined as follows:

Cf.: ***Hemorrhage*** is defined as that which required treatment other than aspiration, compression, and infusion of cold saline.

***Massive hemorrhage*** is defined as blood loss of 300 mL or more, or cases requiring blood transfusion.

Regarding ***pneumonia*** and ***pleurisy***, apparent aggravation of those present before the examination are included.

***Lidocaine intoxication*** is defined as cases which required special treatment for convulsion and loss of consciousness.

***Bronchial asthmatic attack*** is defined as cases which required treatments, such as drip infusion and tracheal intubation.

***Circulatory complications*** include unexpected hypotension, arrhythmia, angina attack, myocardial infarction, and cardiac arrest.

***Respiratory failure*** is defined as cases which required treatments, such as tracheal intubation and artificial ventilation (excluding oxygen administration).

**Number of EBUS-TBNA applications:**

Total number of applications: ( )

Number of applications by objective (overlapping allowed):

Staging of lung cancer: ( )

Definite diagnosis: ( )

(Choose the most likely disease)

Suspected lung cancer: ( )

Suspected malignant lymphoma: ( )

Suspected sarcoidosis: ( )

Others ( ) (details: )

Number of applications by puncture site (overlapping allowed):

Hilar/mediastinal lymph node: ( )

Lesions in the lung field: ( )

Number of applications by operator

Performed by JSRE-accredited non-fellows: ( )

Performed by JSRE-accredited fellows: ( )

**Number of complications**

Total number of complications: ( )

Number of each complication (overlapping allowed):

Hemorrhage: ( )

Massive hemorrhage in the above cases: ( )

Pneumonia: ( )

Pleurisy: ( )

Mediastinitis: ( )

Pericarditis: ( )

Sepsis: ( )

Other infectious complications ( ) (details: )

Pneumothorax: ( )

Cases which required tube drainage in the above: ( )

Lidocaine intoxication: ( )

Bronchial asthmatic attack: ( )

Circulatory complications: ( ) (details: )

Respiratory failure: ( )

Others: ( ) (details: )

Did you encounter damage to an ultrasound bronchoscope and peripheral instruments during the survey period? Yes/No

Number of damaged instruments:

Damage of ultrasound bronchoscope: ( )

Damage of working channels, such as perforation by a needle: ( )

Damage of the fiber region by external compression, such as biting by the patient: ( )

Damage of the ultrasound probe region: ( )

Other damage: ( )

(details: )

Damage of puncture needle: ( )

**Case report of complications**

*Case number ( )*

*Patient background*

1. Sex: male/female
2. Age: ( ) years old
3. Concomitant disease (multiple answers allowed):

lung cancer, pulmonary emphysema, bronchial asthma, chronic heart disease, hypertension, arrhythmia, renal failure, diabetes, others ( )

1. When bronchial asthma is concomitantly present, is pretreatment with a bronchodilator administered: yes/no
2. State of medication with antiplatelet agents and anticoagulants: being treated, withdrawn, not previously medicated
3. Puncture site: hilar mediastinal lymph node, lesions in the lung field

*Background of operator*

1. JSRE-accredited fellow: yes/no
2. Number of EBUS-TBNA applications performed before the occurrence of complications: fewer than 5, 5-19, 20-49, 50 or more
3. Participation in hands-on training: yes/no

*Test conditions*

1. Intravenous sedation: with/without
2. Gauge of needle: 22, 21
3. Number of lymph nodes punctured: 1, 2, 3, 4 or more
4. The maximum short axis of lymph nodes punctured: less than 5 mm, 5-10 mm, larger than 10 mm
5. Punctured site: 1, 2R, 2L, 4R, 4L, 7, 10R, 10L, 11R, 11L, others ( )
6. Echo finding(s) of the punctured site: necrosis was suspected, a cyst was suspected, other regions, unclear
7. Total frequency of punctures: 1, 2, 3, 4, 5 or more
8. On-site cytology: performed/not performed
9. Preventive antibiotic treatment: performed/not performed

*Test results*

1. Possibility of making a diagnosis based on EBUS-TBNA findings: possible/not possible
2. Final diagnosis: lung cancer (non-small cell carcinoma, small cell carcinoma, unclear), metastatic lung tumor, malignant lymphoma, other malignancies, sarcoidosis, other benign diseases, indeterminate

*Complication*

1. Types of complication: hemorrhage (massive hemorrhage), pneumonia, pleurisy, mediastinitis, pericarditis, sepsis, other infectious complications (details: ), pneumothorax, lidocaine intoxication, bronchial asthmatic attack, circulatory complications (unexpected hypotension, arrhythmia, angina attack, myocardial infarction, and cardiac arrest), respiratory failure, others (details: )

Treatment: tube drainage, tracheal intubation, others ( )

Outline of complications (concretely describe the complications that occurred, applied treatment, and the suspected cause to supplement the above).

1. Adverse events resulting from complications:

none in particular, prolongation of hospitalization, treatment to prevent permanent disorder, life-threatening condition, physical disorder, death
